# Cancer Stem Cells Sensitivity Assay (STELLA) in Patients with Advanced Lung and Colorectal Cancer: A Feasibility Study

**DOI:** 10.1371/journal.pone.0125037

**Published:** 2015-05-08

**Authors:** Manolo D’Arcangelo, Matilde Todaro, Jessica Salvini, Antonina Benfante, Maria Luisa Colorito, Armida D’Incecco, Lorenza Landi, Tiziana Apuzzo, Elisa Rossi, Spartaco Sani, Giorgio Stassi, Federico Cappuzzo

**Affiliations:** 1 Istituto Toscano Tumori, Department of Medical Oncology, Civil Hospital, Livorno, Italy; 2 Laboratory of Cellular and Molecular Pathophysiology, University of Palermo, Palermo, Italy; 3 Fondazione Ricerca Traslazionale, Rome, Italy; The First Affiliated Hospital with Nanjing Medical University, CHINA

## Abstract

**Background:**

Cancer stem cells represent a population of immature tumor cells found in most solid tumors. Their peculiar features make them ideal models for studying drug resistance and sensitivity. In this study, we investigated whether cancer stem cells isolation and in vitro sensitivity assay are feasible in a clinical setting.

**Methods:**

Cancer stem cells were isolated from effusions or fresh cancer tissue of 23 patients who progressed after standard therapy failure. Specific culture conditions selected for immature tumor cells that express markers of stemness. These cells were exposed in vitro to chemotherapeutic and targeted agents.

**Results:**

Cancer stem cells were extracted from liver metastases in 6 cases (25%), lung nodules in 2 (8%), lymph node metastases in 3 (12.5%) and pleural/peritoneal/pericardial effusion in 13 (54%). Cancer stem cells were successfully isolated in 15 patients (63%), including 14 with lung cancer (93.3%). A sensitivity assay was successfully performed in 7 patients (30.4%), with a median of 15 drugs/combinations tested (range 5-28) and a median time required for results of 51 days (range 37-95).

**Conclusion:**

The approach used for the STELLA trial allowed isolation of cancer stem cells in a consistent proportion of patients. The low percentage of cases completing the full procedure and the long median time for obtaining results highlights the need for a more efficient procedure.

**Trial Registration:**

ClinalTrials.gov NCT01483001

## Introduction

Cancer is the second cause of death after cardiovascular diseases worldwide and lung, breast, and colorectal tumors are the three major players. Breast (BC) and lung cancer (LC) are the leading cause of cancer death in women and men, respectively, while colorectal cancer (CRC) accounts for approximately 10% of all cancer incidence and mortality [1]. Surgical resection and adjuvant therapy can cure early stage primary tumors. On the other hand, metastatic disease is mostly incurable because of its systemic nature and resistance to therapeutic agents. Indeed, more than 90% of cancer-related deaths can be ascribed to recurrence/relapse and not to primary lesions [2,3]. In the last two decades, treatment outcome has notably improved thanks to a better understanding of cancer biology, the introduction of novel targeted/chemotherapeutic agents and, most importantly, the selection of patients based on biomarkers or tumor-based assays. However, even this novel approach proved to be unsuitable to cure patients with metastatic disease [4–7]. This clinical reality underscores the need to evaluate novel approaches for the treatment of cancer patients and to develop new clinical tools for the selection of the best treatment option for the individual patient.

In recent years, a small subpopulation of undifferentiated cancer cells with stem-like features was identified within tumors and named cancer stem cells (CSC) or tumor initiating cells. They seem responsible for cancer initiation, sustenance, progression and resistance to antineoplastic drugs [8]. According to this hypothesis, CSC originate from the transformation of normal stem cells. Indeed, due to their longevity, stem cells accumulate multiple mutations that are necessary for carcinogenesis. Supporting this hypothesis, normal stem cells and CSC share several important properties, including: *(a)* self-renewal, *(b)* differentiation, *(c)* active telomerase expression, *(d)* activation of anti-apoptotic pathways, *(e)* increased membrane transporter activity and *(f)* ability to migrate and metastasize [9]. Furthermore, cancers usually display extensive phenotypical, functional and molecular heterogeneity that may easily be explained by the CSC theory [10–12]. Two models have been proposed to elucidate the assorted characteristics of cancer cells within a tumor: 1) the clonal evolution model, and 2) the CSC model. While the former postulates that all cells within a cancer have the potential to give rise to new tumors, the latter suggests that only cancer cells with stem-like features can sustain tumor initiation and progression [13–16]. Experimental evidence in a variety of tumors strongly supports the CSC hypothesis. The importance of CSC in tumor initiation has been firmly established in leukemia and recently reported in a variety of solid tumors such as breast, colon, brain, prostate, ovary, lung cancer and melanoma [17–24]. Our group has previously identified and characterized a CD133^+^ tumor cell subpopulation endowed with self-renewal and multi-lineage differentiation capacity just like normal stem cells [25]. This small subpopulation of CSC retains tumorigenic capacity when transplanted into immune-deficient mice [25,26]. The resulting xenografts are histologically and molecularly similar to the parental tumor from which they originated [27]. This intriguing property makes CSC a good model for the study of the biology and sensitivity of tumors of individual patients to antineoplastic agents.

CSC display high expression levels of ATP-binding cassette (ABC) transporters, anti-apoptotic factors, and an active DNA-repair capacity that make them particularly resistant to drugs and toxins [28–30]. In addition, the apparent tumor debulk obtained with chemotherapy contributes to the CSC pool enrichment, thus increasing the risk of developing more resistant phenotypes [31]. This collective evidence suggests that the common inefficacy of conventional therapies on the stem cell population might partially explain cancer resistance to available treatments. Consequently, knowing the spectrum of sensitivity of the CSC subpopulation of the individual tumor and targeting selectively this subpopulation could provide significant improvement of treatment outcomes.

Considering the current lack of specific agents targeting the CSC and the previously reported observations, we conducted a prospective non-randomized study (STELLA trial, ClinicalTrials.gov: NCT01483001) to evaluate whether isolating and testing CSC against a broad spectrum of antineoplastic agents is feasible in the clinical setting. Moreover, the study sought to determine if such an approach could potentially identify drugs with *in vitro* activity against the CSC derived from the individual patient, thus providing new personalized therapeutic options. The study showed that testing of CSC chemosensitivity is feasible in the clinical setting, although a more efficient *in vitro* method is needed.

## Methods

### Eligibility, recruitment and follow up

The protocol for this trial and supporting TREND checklist are available as supporting information; see S1 Protocol and S1 TREND Checklist.

The STELLA study was a prospective, non-randomized, open-label, clinical trial evaluating the feasibility of the intervention of CSC isolation and chemosensitivity assay in clinic (S1 Protocol). The study was approved by the “Ethics Review Board for Investigation of Drugs” of the Civil Hospital of Livorno. Written informed consent was obtained from each patient before enrollment. Patients who were followed or referred to the Medical Oncology Department of the Hospital of Livorno and who met the eligibility criteria were enrolled in the trial. Eligible patients had confirmed cytological or histological diagnosis of metastatic lung, breast or colorectal cancer; pre-treated with standard therapies and without additional standard therapeutic options; with a performance status of 100% according to the Karnofsky score; adequate hematological, renal and liver functions. Main ineligibility criteria included the inability to obtain fresh tumor tissue or neoplastic effusion suitable for CSC extraction and comorbidity potentially interfering with the study. Clinical characteristics of patients as well as molecular features of tumors (when available) were collected and registered in an electronic database in the Department of Medical Oncology of the Civil Hospital of Livorno. Two patients were initially diagnosed with a CRC and included in the study, but a pathology revision showed a different histology (pancreatic cancer and small intestine adenocarcinoma), thus representing a deviation from the study protocol.

The primary objective of the study was to evaluate the feasibility of a sensitivity assay on CSC in a clinical setting, taking into consideration the percentage of patients for whom it was possible to perform the assay and the period of time to obtain the sensitivity results. Secondary end-points were the identification of LC, CRC and BC stem cells and the evaluation of their *in vitro* sensitivity to anti-tumor agents. The latter was expressed as mortality rate, i.e. the percentage of dead cells after the chemosensitivity assay.

Patients were enrolled over a two months period of time (December 6^th^, 2011–January 31^st^, 2012), and were followed up till death or patient’s refusal (December 2011–December 2013) at regular intervals according to clinical need and opinion of the clinician. Telephone interviews were performed in case the patient was unable to attend outpatient visits.

### Tissue Collection, Isolation, and Culture of Cancer Cells

Neoplastic effusion (if present) or tumor tissue from primary cancer or from the most accessible metastatic site was obtained from all eligible patients after informed consent signature in the Civil Hospital of Livorno. Fresh samples were shipped overnight at room temperature to the Cellular and Molecular Pathophysiology Laboratory of the University of Palermo, Italy. Tumor specimens were intensively washed in PBS solution and incubated overnight in Dulbecco’s modified Eagle medium (DMEM, GIBCO) supplemented with penicillin (500 U/ml, GIBCO), streptomycin (500 μg/ml, GIBCO) and amphotericin B (1.25 μg/ml, GIBCO) to avoid microbial contamination. Tumor samples were enzymatically digested with collagenase and hyaluronidase and mechanically shaken for 1 hour at 37°C. Alternatively, neoplastic effusions were separated by centrifugation (1200 rpm, 10 min, 4°C). Collected cells were cultured in stem cell serum free medium supplemented with epidermal growth factor (EGF, 20 ng/mL, Sigma-Aldrich) and basic fibroblast growth factor (bFGF, 10 ng/mL, Sigma-Aldrich) and plated in ultra-low attachment plate (Corning) as previously described [25]. These conditions favour growth of highly tumorigenic stem-like cells, while negatively selecting for less tumorigenic differentiated tumor cells, as previously showed by Eramo et al [24]. Surviving immature tumor cells slowly proliferate and form cell spheres. Sphere-derived cells were expanded by mechanical and/or enzymatic dissociation and re-plated in complete fresh stem cell medium. Cell cultures were maintained at 37°C in a 5% CO_2_ humidified incubator.

The study protocol included *in vivo* experiments that have never been performed because all available cells were used for the chemosensitivity tests in the effort of testing as many drugs as possible and thus identifying a potential treatment for the patient.

### Immunofluorescence characterization of spheres

Sphere-derived cells were proven to be CSC by immunofluorescence characterization for stemness membrane markers. Briefly, tumor spheres were mechanically and enzymatically dissociated to obtain single cells. Cells were fixed and permeablized as previously reported [25]. Cytospun cells were washed in PBS and exposed overnight at 4°C to antibodies against CD133 (AC133, mouse IgG_1_, Miltenyi), CD166 (mouse IgG1, R&D Systems), OCT3/4 (sc-5279, mouse IgG2b, Santa Cruz), ALDH-1 (mouse IgG1, BD Bioscience), CD90 (mouse IgG1k, BD Bioscience), or isotype-matched controls. Afterwards, cells were labelled with fluorescein isothiocyanate (FITC) conjugated mouse antibodies (Invitrogen) and nuclei were counterstained using Toto-3 iodide. The fluorescent staining was evaluated with a confocal microscope.

### In Vitro Sensitivity Assay

After mechanical and enzymatic dissociation, tumor cells were stained with trypan blue and counted with a haemocytometer. Tumor cells (2X10^4^ per well) were exposed to several anti-tumor drugs in order to evaluate their sensitivity to the therapeutic compounds. Table 1 indicates the drugs or combinations used. Clinicians empirically chose the drugs to test on the basis of the previous treatment regimens and the basic molecular aberration of the individual patient. The *in vitro* sensitivity assay was performed simultaneously in two 96-well ultra-low attachment plates (Corning). The treatment time was determined based on drugs half-life and continued up to 96 hours. Each regimen was evaluated in triplicate.

**Table 1:** Drugs and combinations tested *in vitro*.

Chemotherapeutic agent	Target agent
1. Docetaxel	24. Afatinib
2. Temozolomide	25. Cetuximab
3. Topotecan	26. Crizotinib
	27. Erlotinib
4. Epirubicin + paclitaxel	28. Nilotinib
5. Carboplatin + etoposide	29. Sunitinib
6. Carboplatin + pemetrexed	30. Sorafenib
7. Carboplatin + vinorelbine	31. Trastuzumab
8. Carboplatin + vinorelbine + cetuximab	
9. Cisplatin + docetaxel	32. Afatinib + cetuximab
10. Cisplatin + etoposide	33. Crizotinib + Pemetrexed
11. Cisplatin + pemetrexed	34. Crizotinib + cetuximab
12. Cyclophosphamide + adriamycin + etoposide	35. Crizotinib + everolimus
13. Epirubicin + etoposide	36. Crizotinib + etoposide
14. Epirubicin + ifosfamide	37. Erlotinib + cetuximab
15. Gemcitabine + trastuzumab	38. Erlotinib + crizotinib + cetuximab
16. Oxaliplatin + gemcitabine	39. Erlotinib + everolimus
17. Oxaliplatin + paclitaxel	40. Erlotinib + crizotinib
18. Paclitaxel + trastuzumab	41. Erlotinib + trastuzumab
19. Pemetrexed + topotecan	42. Everolimus + paclitaxel
20. Pemetrexed + cetuximab	43. Nilotinib + erlotinib
21. Vinorelbine + trastuzumab	44. Nilotinib + crizotinib
22. 5-fluorouracyl + oxaliplatin	45 Sorafenib + paclitaxel
23. 5-fluorouracyl + irinotecan	46. Sunitinib + paclitaxel
	47. Sunitinib + erlotinib

Cell viability was evaluated with orange acridine/ethidium bromide (AO/EB) staining, as previously described [34]. The cell suspension was incubated in AO/EB solution in 1:1 ratio respectively and gently mixed. This procedure was performed just before quantification with a confocal microscope. Eight μl of cell suspension were placed onto a microscopic slide and covered with a glass coverslip. A confocal microscope was used to examine the sample and quantify cell death [34]. The results of the sensitivity assay were communicated to clinicians in order to select, when possible, a therapy regimen optimized for the individual patient.

### Statistical Analysis

Sample size was computed with the software IBM SPSS Sample Power 3.0 on the basis of the percentage of patients for whom it was possible to perform the assay. Using the test for proportion (two sided test), under the assumption of a percentage of 33% (versus standard procedure of 5%), a sample size of 18 patients was required, with a power of 90% and a significance level of 0.05. Descriptive statistics (frequency, percentage, median and inter-quartile range) were used to analyse data where appropriate. Results of the *in vitro* procedure (type of samples collected, CSC isolation failure rate) were analysed considering the total number of samples. The other results (sensitivity assay success rate, time for result and number of drugs tested) were analysed considering single patient data of the “intention to treat” population.

## Results

### Patients characteristics

**Fig 1:**
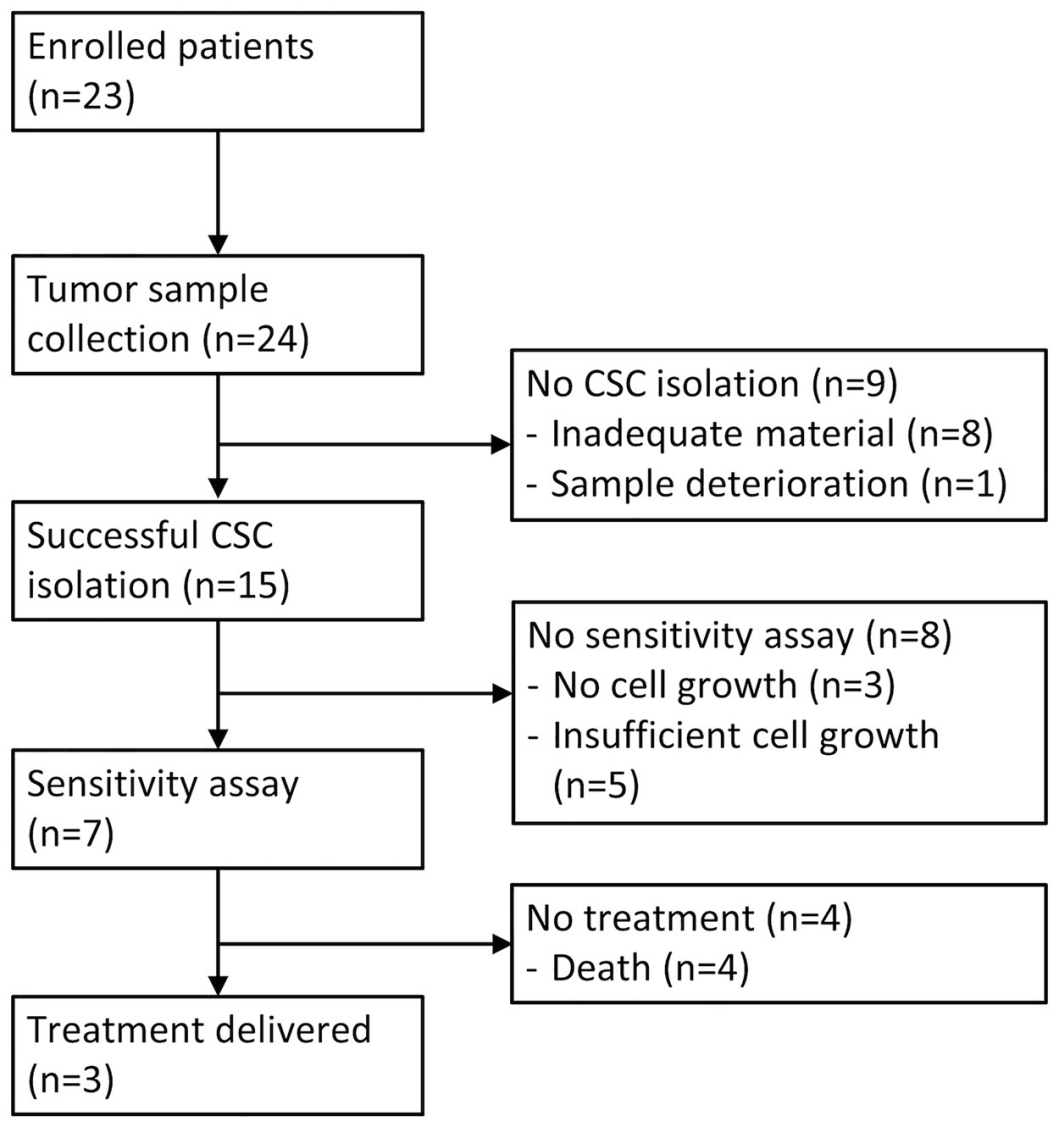
Flow chart of the study.

From December 6^th^ 2011 to January 31^st^ 2012, a total of 23 patients were identified at the Medical Oncology Department of Livorno and enrolled in the study (Fig 1). All screened patients were eligible and started the procedure for CSC isolation. All patients were included in the following analyses. Table 2 summarizes the clinical characteristics of individual patients. Median age was 66 years (interquartile range, IQR: 51–72) and the majority of subjects were male (n = 15, 65%) and had lung cancer (n = 18, 78%). No BC patient was enrolled. Bio-molecular characterization was not available in 5 cases. Among lung cancer patients, *EGFR* mutations were detected in 4 cases, *ALK* translocation in 1 case and *KRAS* mutation in another case. Two of the three CRC patients harboured a *KRAS* mutation. Patients received a median of 3 previous therapy lines (IQR: 1–3). All patients completed the follow-up, except for one patient that is still alive. No patient was lost to follow-up.

**Table 2:** Individual patients characteristics.

Patient ID	Age	Sex	Primary Cancer	Histology	Molecular aberration	Previous treatment lines	Smoke habit	Number of tissue/effusions collections
**NP001**	72	F	LC	SCLC	No	1	PS	1
**PM002**	49	M	CRC	ADC	KRAS mut	3	NV	1
**SN003**	69	F	LC	ADC	No	3	NV	1
**LG004**	54	M	LC	SqC	No	2	PS	1
**ZL005**	45	F	LC	ADC	EGFR mut	3	NS	2
**GD006**	42	M	LC	ADC	EGFR mut	7	PS	1
**RG007**	71	M	LC	ADC	No	4	CS	1
**TR008**	70	F	LC	ADC	No	4	NS	1
**CM009**	48	F	LC	ADC	ALK transl	1	NS	1
**GA010**	66	M	LC	NAS	No	0	PS	1
**CN011**	56	M	CRC	ADC	KRAS mut	4	PS	1
**BU012**	80	M	LC	ADC	No	1	NS	1
**BG013**	71	M	GI	ADC	NE	0	PS	1
**RR014**	73	M	GI	ADC	NE	1	PS	1
**CM015**	74	M	LC	ADC	EGFR mut	2	PS	1
**LC016**	56	M	LC	ADC	No	2	PS	1
**RE017**	73	F	CRC	ADC	NE	1	NS	1
**SL018**	50	F	LC	ADC	No	3	NS	1
**PD019**	52	M	LC	ADC	KRAS mut	0	PS	1
**CV020**	49	M	LC	SqC	NE	2	PS	1
**ME021**	85	F	LC	ADC	No	1	NS	1
**CR022**	55	M	LC	SqC	NE	6	PS	1
**SF023**	67	F	LC	ADC	EGFR mut	3	NS	1

Abbreviations: F, female; M, male; LC, lung cancer; CRC, colorectal cancer; GI; other gastrointestinal cancer; SCLC, small cell lung cancer; ADC, adenocarcinoma; SqC, squamous cell carcinoma; PS, previous smoker; NV, never smoker; CR, current smoker; NE, not evaluated.

### Cancer stem cells isolation and sensitivity assay

Tumor biopsy or neoplastic effusion collection procedure was repeated no more than one time per patient, except in one case. There were no complications resulting from the tumor tissue/effusion collection procedure. Isolation of CSC was performed using pleural/peritoneal/pericardial effusion in 13 cases (54%), tumor tissue from lung metastases in 2 cases (8%), liver metastases in 6 (25%) and lymph node metastases in 3 (13%). CSC were successfully isolated in 15 out of the 24 tumor samples collected (63%). Material was not adequate in 8 cases and one specimen deteriorated during transportation. The failure rate was higher with tumor biopsies (5 failed cases out of 11, failure rate: 45%) than with malignant effusions (3 failed cases out of 13, failure rate: 23%). In particular, the lowest yield was obtained with lymph node metastases (2 failed cases out of 3, failure rate: 67%). According to the primary site of cancer, failure rate was 30% for lung cancer and 80% for gastrointestinal tumors.

**Fig 2:**
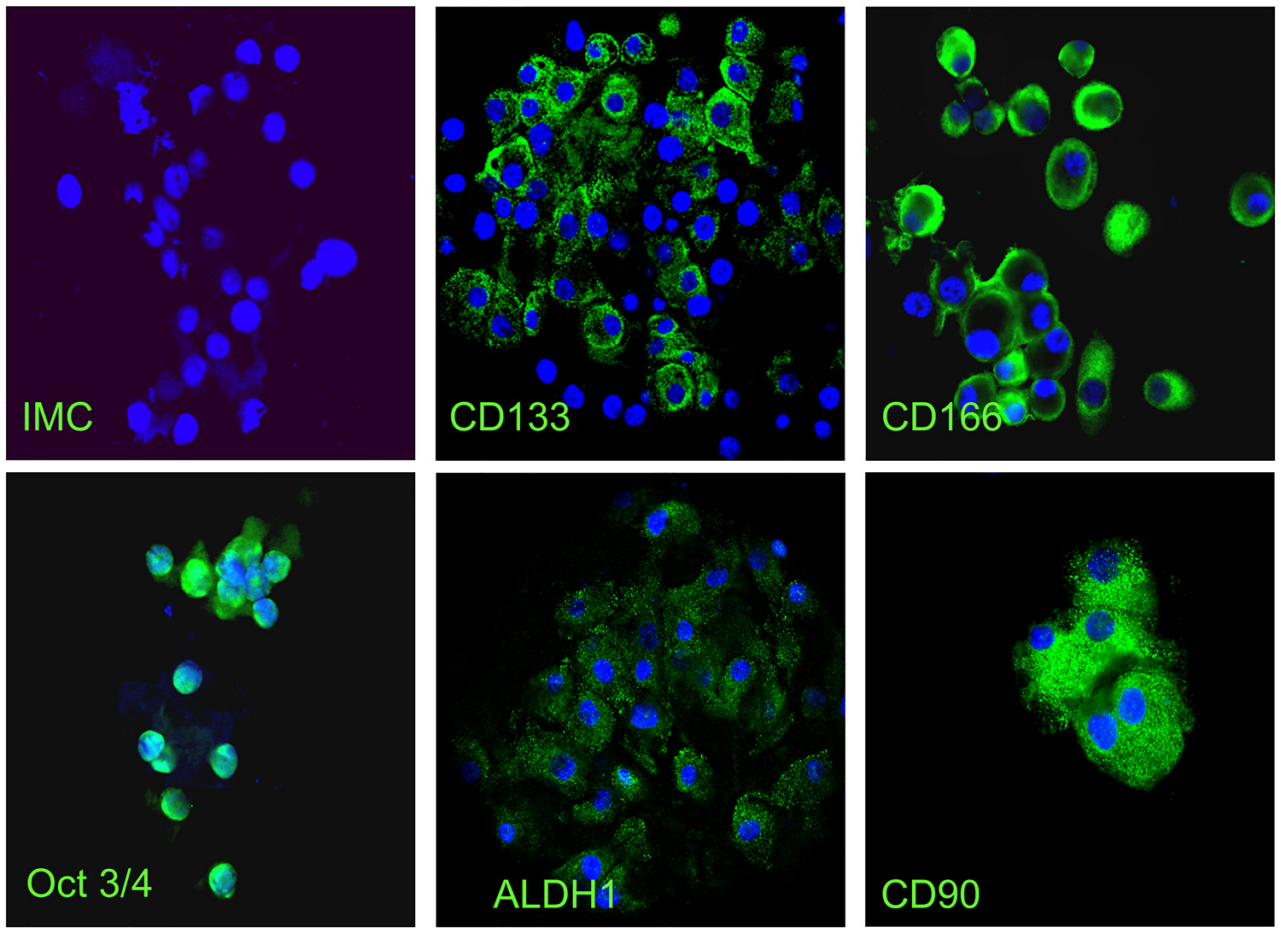
Phenotypical characterization of immature tumor cells isolated from tumor samples with immunofluorescence assay. The following panel of images refers to the characterization of cells isolated from the sample of a patient enrolled in the study. Green labelling is typical of positive cells. All markers of stemness (CD133, CD166, Oct 3/4, ALDH1, CD90) are positive in this CSC sample.

Freshly isolated lung tumor cells had stem-like phenotype confirmed by the concomitant expression of the most common stemness markers, including CD133, CD166, OCT3/4, ALDH-1 and CD90 (Fig 2) [32,33]. The concomitant high expression of these markers strongly supports the fact that the isolated cells were CSC. Table 3 shows the pattern of expression of the markers in the cases in which a sensitivity assay was performed. No immuno-fluorescent study was performed on gastro-intestinal samples for limited number of isolated cells and insufficient *in vitro* growth.

**Table 3:** Sensitivity assay results and patients treatment.

	SN003	LG004	GD006	TR008	GA010	PD019	CV020
**Marker**							
CD133	55%	60%	70%	60%	65%	55%	70%
CD166	70%	80%	80%	85%	70%	80%	65%
Oct 3/4	70%	75%	75%	70%	80%	75%	80%
ALDH1	90%	90%	80%	80%	70%	90%	80%
CD90	90%	90%	90%	90%	70%	90%	80%
**Time to results** (days)	37	52	95	51	44	56	50
**Tested drugs** (n)	5	5	5	26	26	15	18
**Drugs determining mortality rate** (code)							
<30%	2,4,5,17,33	8,12,14,15,19	11,24,28,32	1,3,7,8,9,10,12,14,17,18,21,22,23,24,29,30,32,37,39,42,45,46	5,7,9,10,12,14,17,18,21,22,23,24,26,29,30,32,33,37,38,39,42,45,46	1,6,17,18,24,25,26,27,28,29,30,31,34,40,43	3,5,6,13,16,17,18,24,26,27,28,29,30,35,41,44,47
31–50%	0	0	17	5,25,32	1,3,8	0	36
51–75%	0	0	0	0	0	0	0
76–90%	0	0	0	38	0	0	0
>90%	0	0	0	0	0	0	0
**Treatment** (yes,no/drug code)	Yes/17	No/NA	Yes/17	No/NA	No/NA	Yes/6	No/NA
**Response to therapy**	PD	NA	SD	NA	NA	PD	NA

Abbreviations: PD, progressive disease; NA, not applicable; SD, stable disease.

Drugs and combinations are codified as in Table 1.

**Fig 3:**
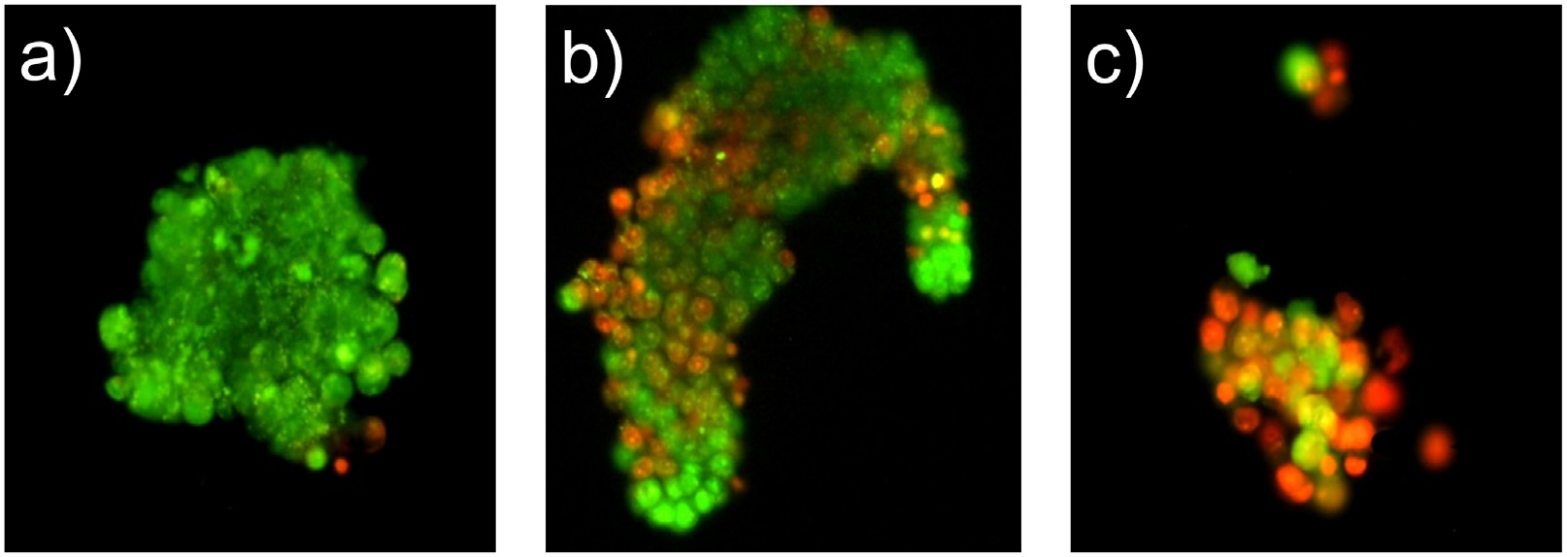
Evaluation of cell mortality with acridine orange/ethidium bromide (AO/EB) staining after exposition to chemotherapeutic agents. AO is a vital dye and stains both live and dead cells. EB penetrates into cells with disrupted cytoplasmic membrane, staining only dead cells. Therefore, viable cells appear uniformly green and dead cells are labelled in orange. a) Low sensitivity (cell mortality 5%). b) Average sensitivity (cell mortality 40%). c) High sensitivity (cell mortality 80%).

Among the 15 cases in which CSC were successfully isolated, *in vitro* cell growth was observed in 12 and only in 7 cases the number of available cells was sufficient to perform a chemosensitivity test. The overall success rate of the whole procedure was 30.4%. The *in vitro* response to treatment was evaluated in 96 wells plates in triplicate with AO/EB staining. The AO/EB staining was used to visualize nuclear changes and apoptotic body formation that are characteristic of apoptosis. Whereas AO is a vital dye and stains both live and dead cells, EB stains only cells that lost membrane integrity. Therefore, viable cells appeared uniformly green and dead cells incorporated EB and, consequently, were stained in orange (Fig 3). Response to treatment, observed in the first plate labelled with AO/EB, was homogeneous in all three wells. Viable cells of the second plate were kept in culture for observation and no morphologic or phenotypical change was detected.

All patients whose tumors were successfully tested *in vitro* had LC, including 5 lung adenocarcinomas, 1 undifferentiated non-small cell lung cancer and 1 small cell lung cancer. The median time between sample collection and sensitivity assay results was 51 days (IQR: 47–54). Table 1 indicates the drugs or combinations used for the *in vitro* sensitivity assay and more detailed information about the single tests can be found in Table 3. The median number of drugs/combinations that were tested was 15 (IQR: 5–22). The *in vitro* CSC mortality rate ranged from 0 to 80%, but only in one case the mortality rate was >50% (Table 3).

### Patients treatment

The study did not contemplate the treatment of patients and its endpoints did not include whether the *in vitro* results correlate with the clinical response. However no valid therapeutic option was available for these patients and, when possible, we offered them a treatment with one of the tested drugs. The chosen regimen was always the one with the highest *in vitro* mortality rate. As there is no recognized cut-off value of the *in vitro* result defining clinical sensitivity/resistance, it cannot be ruled out that regimens with low *in vitro* mortality may produce clinical responses and, therefore, the chosen regimen could have had a low mortality rate. As shown in Table 3, among the 7 subjects for whom a sensitivity assay was available, only 3 received a treatment based on the test results. The highest mortality rate observed *in vitro* was 10% in 2 of these patients and the disease progressed soon after the start of treatment. The first patient was a never smoker woman with a metastatic lung adenocarcinoma with no molecular driver aberration. She received a combination of oxaliplatin and paclitaxel, but the disease progressed after only two cycles. The second patient was a former smoker man with *KRAS* mutated advanced adenocarcinoma of the lung. He received a combination of carboplatin and pemetrexed and his disease progressed soon after. On the other hand, in a young man with a metastatic *EGFR* mutated adenocarcinoma of the lung the *in vitro* assay showed a cell mortality of 40% for the combination of oxaliplatin and paclitaxel. The patient received 5 cycles of the above-mentioned chemotherapeutic drugs, achieving a stabilization of disease. Despite the beneficial effect, considering that the patient had already received 7 lines of treatment, the clinician decided to stop treatment for increased risk of toxicity. The other 4 patients for whom a sensitivity test was performed did not receive the treatment because they passed away before results became available. Overall, these anecdotal findings seem to indicate that higher *in vitro* sensitivity could translate into better clinical outcome.

## Discussion

Accumulating evidence supports the existence of CSCs in human tumors. These cells possess the capacity to self-renew and differentiate into mature tumor cells. They seem to drive tumor initiation, progression, and metastasis. Given their high resistance to anticancer drugs, they are believed to substantially contribute to relapse after chemotherapy and represent an attractive therapeutic target to potentially achieve long lasting therapeutic responses. Therefore, the recognition of the most active drugs on the CSC subpopulation has the potential to identify the best treatment choice for the individual patient or new treatment options for patients progressing on standard therapies.

Previous studies have already described the isolation procedure of CSC. The STELLA study is the first prospective study that evaluates the applicability of this procedure in a clinical setting with the aim of testing the *in vitro* sensitivity of CSC and potentially identifying new therapeutic options for the patient. Considering that feasibility was the primary end-point of the study, a limited number of individuals was enrolled, the vast majority with a diagnosis of LC, the most frequent disease referred to our Institution. The study showed that the procedure was feasible in 65% of cases, leading to a chemo-sensitivity result in one third of patients in a median time of 51 days. Overall, this preliminary experience gave us very relevant information.

First of all, site of tumor sample collection was critical for the success of the isolation procedure since neoplastic effusions were the most proficient for such purpose. This is also a relevant aspect for clinical practice since effusion collection is relatively easy and often required for symptom management, it has low risk and it is generally well accepted by patients. In our opinion the sample type, whether it was fresh biopsy tissue or neoplastic effusion, was the factor that mainly impacted on the low percentage of patients benefitting from the test. Other factors, such as the type of culture medium, may have had a minor impact.

The median time between sample collection and sensitivity assay results was 51 days. This is a relatively long period of time for metastatic cancer patients, explaining why less than 50% of subjects for whom the sensitivity test was available received a therapy based on its results. Whether this lapse of time is sufficient to further alter the tumor cell biology and their sensitivity to drugs is not known and currently there is no scientific evidence of this. In order to shorten this time frame, based on the STELLA experience, an ongoing clinical study (LUCAS) is using a different assay (Tecan Infinite F500 after Cell Titer-Glo Luminescent Cell Viability Assay; Promega). This method determines the number of viable cells based on quantification of a luminescent signal that is proportional to the amount of ATP; it requires a smaller number of cells and delivers actionable results in only 7 days from the biopsy. This might help achieve results in a shorter period of time and test more drugs for patients with slow *in vitro* growth of CSC. Moreover, it delivers quantitative results, eliminating the inferior precision of the previous semi-quantitative method. This new approach could also improve the percentage of patients in which the chemosensitivity test can be successfully performed. In fact, in the STELLA study the sensitivity assay was performed in only 7 cases mainly for the poor *in vitro* cell growth.

In our study CSC showed high resistance to currently available drugs, including standard chemotherapy agents and targeted therapies, with only one case showing a mortality rate above 50%. This is not unexpected considering the high resistance of CSC to commonly used antitumor agents, highlighting the urgent need for more effective drugs specifically targeting this cell population.

In conclusion, the approach used for the STELLA trial allowed CSC isolation in a consistent proportion of patients. The low percentage of cases completing the full procedure of chemosensitivity assay and the long median time for obtaining the full results highlights the need for a more efficient procedure. In such perspective, the currently ongoing LUCAS study will clarify the potential clinical implications of therapies targeted on CSCs.

## Supporting Information

S1 Protocol Study protocol, information letter for general practitioner and informed consent form. (PDF)

S1 TREND Checklist TREND checklist. (PDF)
