# Supplementary material for: Cancer Stem Cells Sensitivity Assay (STELLA) in Patients with Advanced Lung and Colorectal Cancer: A Feasibility Study
Source: PLoS One. 2015 May 8;10(5):e0125037. doi: 10.1371/journal.pone.0125037 (PMC4425502; doi:10.1371/journal.pone.0125037)
Supplement: S1 Protocol — (PDF) [file pone.0125037.s001.pdf]

STELLA: A Feasibility Study on Stem Cells Sensitivity Assay

**Codice studio:**	LIVONCO 2011/001
**Codice EudraCT:**	2011-003421-10
**Promotore:**	Associazione Oncologia Traslazionale<br>*Via Giovanni Pacini 12*<br>*90138 Palermo*
**Coordinatore:**	Dr. Federico Cappuzzo<br>Dipartimento di Oncologia<br>Istituto Toscano Tumori-Ospedale Civile<br>*Viale Alfieri 36*<br>*57100 Livorno*
**Investigatori Principali:**	Dr. Federico Cappuzzo<br>Dipartimento di Oncologia<br>Istituto Toscano Tumori-Ospedale Civile<br>*Viale Alfieri 36*<br>*57100 Livorno*<br><br>Prof. Giorgio Stassi<br>Laboratorio fisiopatologia cellulare e molecolare<br>Università di Palermo<br>Via Liborio Giuffre', 5<br>90127 Palermo
**Partecipanti:**	Dr.ssa Matile Todaro<br>Dr.ssa Lorenza Landi<br>Dr. Manolo D'Arcangelo<br>Dr. Gabriele Minuti<br>Dr . Spartaco Sani<br>Dr . Gianfranco Menconi
**Infermiera di ricerca**	Dr.ssa Jessica Salvini
**Data management**	Dr.ssa Elisa Lani
**Statistico**	Dr.ssa Elisa Rossi

VERSION / DATA: ***03/*27 ottobre 2011**

INDEX

**TRIAL SYNOPSIS**	**page 3**
**Abstract**	**page 6**
**1.0 – BACKGROUND**	**page 7**
**2.0 – RATIONALE**	**page 9**
**3.0 – STUDY OBJECTIVES**	**page 11**
**3.1 - Primary end-point**	**page 11**
**3.2 - Secondary end-points**	**page 11**
**4.0 – PATIENTS SELECTION CRITERIA**	**page 11**
**4.1 - Inclusion Criteria**	**page 11**
**4.2 - Exclusion Criteria**	**page12**
**5.0 – STUDY DESIGN**	**page 12**
**6.0 – EXPERIMENTAL PROCEDURES**	**page 12**
**6.1 – Patients**	**page 12**
**6.2 - LC, CRC and BC CSCs identification**	**page 13**
**6.3 - Preclinical model**	**page 15**
**6.4 - Tumor sensitivity to anti-tumoral agents**	**page 16**
**7.0 – PARTECIPATING CENTERS**	**page 16**
**8.0 – STUDY DURATION**	**page 16**
**BIBLIOGRAPHY**	**page 17**
**APPENDIX I - Karnofsky performance status scale**	**page 26**
**APPENDIX II – Patient information letter and informed consent form**	**page 28**
**APPENDIX III – GP information letter**	**page 31**

Trial synopsis

**TRIAL SYNOPSIS**	**STELLA: A FEASIBILITY STUDY ON THE STEM CELLS SENSITIVITY ASSAY**
**PROTOCOL VERSION**	Version 3.0 – 27 October 2011
**SPONSOR**	Associazione Oncologia Traslazionale (A.O.T.)
**BACKGROUND AND RATIONALE**	Lung Cancer (LC), colorectal cancer (CRC) and breast cancer (BC) are the major killers in oncology, accounting for about 40% of cancer deaths. Although progresses have been made in the last few years, unfortunately no patient with metastatic disease can obtain a definitive cure.<br>A recent hypothesis is that cancer is driven by a small subpopulation of cells called “cancer stem cells” (CSCs) or “tumor initiating cells” with an unlimited proliferative potential and the ability to reproduce the original human tumor in experimental animal models. These cells are thought to be responsible for the development of the tumor and represent the only cell population able to sustain tumor growth and progression. Therefore, CSCs represent the elective target for new targeted therapies, endowed with high and selective toxicity towards the tumor but harmless towards normal cells.<br>Current technologies allow us to isolate and expand in vitro the CSCs from tumor specimens, testing their sensitivity to different anticancer drugs in a short period of time. Therefore, there is the potential opportunity to identify and offer an individualized therapy to LC, CRC and BC patients.
**STUDY END-POINTS**	**Primary:**<br>- To evaluate the feasibility of the project<br><br>**Secondary:**<br>- To identify LC, CRC and BC stem cells<br>- To investigate the sensitivity of LC, CRC and BC stem cells to anti-tumor agents *in vitro* and in xenograft models<br>- To identify drugs potentially effective for a specific patient

**INCLUSION CRITERIA**	- Histologically/cytologically confirmed diagnosis of metastatic LC, CRC or BC<br>- Availability of tumor tissue suitable for CSCs extraction<br>- Performance status of 100% according to Karnofsky score<br>- Failure of conventional therapies or no therapy of proven efficacy<br>- Adequate hematological, renal and liver functions<br>- No concomitant comorbidity potentially interfering with the study<br>- Informed consent form signature.
**EXCLUSION CRITERIA**	- Performance status <100% according to Karnofsky score<br>- Patient suitable for standard therapies<br>- Important comorbidity interfering with the study<br>- Significant alteration of liver, hematological or renal function(s)<br>- No informed consent form signature
**STUDY DESIGN**	This is a prospective study assessing feasibility of CSCs isolation in LC, CRC and BC.<br><br>Patients with a previously performed diagnosis of LC, colon cancer or breast cancer with no further standard therapy options, with a Karnofsky performance status of 100% and with tumor tissue available will be considered eligible for the study. Tumor tissue will be collected before study entry, i.e tissue obtained during a diagnostic or therapeutical procedure, like surgery or biopsies with other purposes than the protocol. After CSCs isolation and culture, *in vitro* tumor sensitivity to chemotherapy drugs will be tested on tumor cell cultures per each patient. By using cancer spheres we will also generate orthotopic xenograft models that recapitulate the parental tumor behaviour, including the aggressive features and the invasiveness potential. Orthotopic injection technique will be assessed in 5 weeks-old NOD/SCID mice.
**LABORATORY PROCEDURES**	After antibiotics and antifungine treatment tumor specimens will be dissociated by enzymatic digestion and recovered cells cultured in serum-free medium. These culture conditions select for immature tumour cells, while non malignant or differentiated cells are negatively selected. Surviving immature tumor cells slowly proliferate giving rise to tumour cell aggregates, “spheres”. Sphere-forming cells can be expanded by mechanical dissociation of spheres, followed by re-plating of single cells and residual small cell aggregates in complete fresh medium.<br>Differentiation of LC, CRC and BC cancer sphere-forming cells will be obtained by cell culture in LC, CRC and BC cell-specific medium (Cambrex). Phenotype of LC, CRC and BC spheres and their differentiated progeny will be analyzed by flow cytometric analysis or immunofluorescence. In particular stem cell markers such as CD133, CD34 and BCRP1 will be analyzed.<br>Successively, LC, CRC and BC spheres will be analyzed in order to define the status of pathways involved in the process of proliferation, self-renewal and survival.

**LABORATORY PROCEDURES**	By using LC, CRC and BC spheres we will generate orthotopic xenograft models that recapitulate the parental tumor behaviour, including the aggressive features and the invasiveness potential.<br>Infection of LC, CRC and BC CSCs with lentiviral vector, coding for green fluorescent (GFP), as well as luciferase reporter proteins, will allow CSCs tracking in vivo. The local tumor and the invasiveness development will be monitored through whole-body imaging techniques, that will permit to detect, localize and quantify dynamically the optical signal - bioluminescence - in a non invasive localization of the marked cell population. Once we are sure of the success of tumour growing, mice will be sacrificed. Tumor will be removed for morphological characterization and phosphoproteomic analysis. This latter will be performed through reverse phase protein microarray.<br>To selectively discriminate the effective therapeutic compounds against the putative tumor and invasion initiating cells, we will measure the viability of clonogenic LC, CRC and BC after the exposure to several anti-tumor drugs differentially combined at singular [illegible]
**STUDY DURATION**	Enrollment will begin after local Ethical Committee approval. The study will be completed in 6 months, starting on November 2011. A total of 20 patients will be enrolled.
**CENTERS**	- Medical Oncology Division of Civil Hospital of Livorno<br>- Fondazione Maugeri of Pavia and Laboratory of Cellular and Molecular Pathophysiology of Palermo for laboratory procedures
**PATIENTS REGISTRATION**	Patients registration will be centralized at the Clinical Trials Office of Medical Oncology Division of Livorno. A total of 20 patients will be enrolled.
**DATA MANAGEMENT**	The data center will be the Clinical Trials Office of Medical Oncology Division of Livorno Civil Hospital which can be contacted at the following phone number 0586 223189 or via fax 0586223457

Abstract

Lung cancer (LC), colon-rectal cancer (CRC) and breast cancer (BC) are considered the biggest killers in oncology, counting for about 40% of cancer deaths. During the last decade, improvement in cancer biology knowledge led to discovery and clinical use of new agents specifically targeting proteins critically involved in cancer growth. Although these new agents, including the Epidermal Growth Factor Receptor Tyrosine Kinase Inhibitors (EGFR-TKIs), the anti-EGFR antibodies (cetuximab, panitumumab), the anti-HER2 antibody (trastuzumab) and the anti vascular endothelial growth factor (VEGF) antibody (bevacizumab), are significantly contributing to increase duration of life, no patient with metastatic disease can obtain a definitive cure.

It has long been known that tumors, although clonal by origin, are rather not homogeneous in their cellular composition. The existence of different degrees of differentiation would be caused by a cellular population with the self-renewal, undifferentiated state and multipotency stem cell-like features. This results in the hypothesis that cancer is driven by a small subpopulation of cells called “cancer stem cells” (CSCs) or “tumor initiating cells” with an unlimited proliferative potential and the ability to reproduce the original human tumor in experimental animal models. This small population is suggested to be responsible for tumor initiation, progression and spreading. CSCs are believed to be the result of acquired epigenetic and genetic changes that can change signaling pathways controlling proliferation, differentiation and apoptosis. Such mutations would be passed on to all of the stem cells' progeny, allowing evolution towards malignancy. The possibility to isolate and study CSCs represents a revolutionary approach in cancer research. Indeed, these cells are responsible for the development of the tumor and represent the only cell population able to sustain tumor growth and progression. Therefore, CSCs represent the elective target for new targeted therapies, endowed with high and selective toxicity towards the tumor but harmless towards normal cells.

Aim of the present study is to assess the feasibility of isolating CSCs from LC, CRC and BC patients not suitable for conventional therapies, offering them a new potentially effective treatment. For this study, patients with histologically/cytological proven LC, CRC and BC in treatment with the last approved anticancer agent, with no further standard therapeutic options and with a good performance status will be included. Isolation and characterization of CSCs will be made starting from samples of tumor tissue surgically or biopsy obtained from patients with LC, CRC or BC. By using cancer cell spheres, orthotopic xenograft models will be generated. To selectively discriminate the effective therapeutic compounds against the putative tumor and invasion initiating cells, we will measure the viability of clonogenic LC, CRC or BC after the exposure to several anti-tumor drugs differentially combined at singular time point up to 96 hours.

The study will be conducted in a cohort of 20 LC, CRC and BC patients. The primary end-point is feasibility. Secondary end-points include identification of LC, CRC and BC stem cells and sensitivity of LC, CRC and BC stem cells to anti-tumor agents *in vitro* and in xenograft models.

1.0-BACKGROUND

In 2011, Lung Cancer (LC), colorectal cancer (CRC) and breast cancer (BC) remain the leading causes of cancer-related death worldwide [1]. For patients with metastatic disease definitive cure is not achievable and median survival is approximately 1 year for NSCLC and about 2 years for metastatic CRC and BC [2]. For NSCLC patients, chemotherapy with third-generation platinum-based doublets represented the standard of care until recently, when major breakthroughs in the knowledge of cancer biology has granted the signaling out of numerous targeted therapies for NSCLC treatment. Large phase III clinical trials demonstrated that a proper front-line therapy of a patient with metastatic NSCLC should de based on tumor histology and biology. Patients harboring activating Epidermal Growth Factor Receptor (EGFR) mutations benefit more from EGFR-Tyrosine Kinase Inhibitors (EGFR-TKIs) than from standard platinum-based chemotherapy at least in terms of response rate, progression-free survival (PFS) toxicity profile and quality of life [3-7]. Although no phase III data are currently available, patients with ALK translocation seem to derive a substantial and sustained benefit when treated with crizotinib, an oral c-MET and ALK inhibitor [8]. In patients without any detectable specific target, histology is the major factor influencing therapy choice. Patients with non-squamous histology seem to benefit more from a pemetrexed-based chemotherapy [9], while in squamous histotype, the classical combination of platin (cisplatin or carboplatin) together with gemcitabine, vinorelbine or a taxane, remains the standard of care [2]. At the present time there are only three agents approved for second-line therapy, including pemetrexed, docetaxel and erlotinib, that is also the only drug approved for third-line therapy. These three agents are considered equally effective in unselected patients, with a toxicity profile in favor of erlotinib and pemetrexed [10,11].

For CRC patients, over the past 2 decades the repertoire of chemotherapeutic agents has increased and extended median overall survival to more than 20 months. Today the active drugs for CRC include 5-Fluorouracyl, irinotecan, oxaliplatin and mytomicin C. An increasing body of evidence also supports the addition of targeted agents, those directed towards VEGF (bevacizumab) and EGFR (cetuximab, panitumumab), to expand treatment options for patients with metastatic disease. Bevacizumab, added to a 5-fluorouracil (5FU) ± irinotecan-based chemotherapy as first-line treatment, has been shown to improve response rates and survival of mCRC patients when compared

to the chemotherapy alone [12-14]. An improvement of PFS was also shown in first-line with the addition of bevacizumab to oxaliplatin-based chemotherapy [15]. A randomized phase III study also reported a clinical efficacy of the association of bevacizumab and FOLFOX4 as second-line in mCRC patients previously treated with a fluoropyrimidine and irinotecan, with a significant improvement in response rates, PFS and OS when compared to FOLFOX4 alone [16]. Moreover, the benefits of cetuximab in metastatic colorectal cancer are well documented in clinical trials. There is evidence of the role of cetuximab not only in irinotecan-refractory or heavily pretreated patients, but also of the efficacy and safety of the addition of this agent to FOLFIRI (irinotecan/5-fluorouracil/leucovorin) in first-line metastatic colorectal cancer, with an enhanced effect in patients with KRAS wild-type tumors [17]. In these patients, a recent meta-analysis of the pooled Cetuximab Combined with Irinotecan in First-Line Therapy for Metastatic Colorectal Cancer (CRYSTAL) and Oxaliplatin and Cetuximab in First-Line Treatment of mCRC (OPUS) patient populations confirms that the addition of cetuximab to first-line chemotherapy achieves a statistically significant improvement in the best overall response, overall survival time, and progression-free survival (PSF) compared with chemotherapy alone [18].

In metastatic BC several options are available. In HER-2 positive patients anti-HER2 strategies, based on the use of trastuzumab and lapatinib, can prolong patients life expectancy. Several phase III trials have demonstrated an improvement in terms of overall response rate, progression free survival and overall survival if these drugs are used in association to chemotherapy [19, 20]. In HER2 negative hormone-sensitive patients anti-hormonal drugs, both steroidal and non-steroidal, are available and their efficacy have been widely demonstrated [21]. In triple-negative patients recent trials have suggested a benefit when a PARP-inhibitor (olaparib) is associated to chemotherapy [22]. Several chemotherapeutic agents have demonstrated to be active in metastatic BC: antracyclines, fluoropyrimidines, taxanes and vinca alkaloids, even if none of them has been demonstrated to improve overall survival. On the other hand, the role of bevacizumab is not definitively clarified: discordant results on survival data from the phase III trials have brought recently to withdrawal of bevacizumab approval from FDA [23].

Although many treatment options are available for these cancer patients, none of the above mentioned drugs is able to cure any patient. Invariably, all patients relapse and die for their disease,

clearly indicating that our therapies are able to eradicate only a part of the tumor, the sensitive phenotype.

2.0-RATIONALE

It has long been known that tumours, although clonal by origin, are rather not homogeneous in their cellular composition. The existence of different degrees of differentiation would be caused by a cellular population with the self-renewal, undifferentiated state and multipotency stem cell-like features [24]. Studies published on major international scientific journals demonstrated that tumors are spread and become resistant to therapy because of a subpopulation of stem cells, characterized by features of self-renewal and multi-potentiality. Evidence is accumulating that part of the aberrant differentiation in cancers is due to a remnant response of the tumor cells to differentiating signals. This results in the hypothesis that cancer is driven by a small subpopulation of cells called “cancer stem cells” (CSCs) or “tumor initiating cells” which possess an unlimitate proliferative potential and the ability to reproduce the original human tumor in experimental animal models. This small population is suggested to be responsible for tumor initiation, progression and spreading. CSCs are believed to be the result of acquired epigenetic and genetic changes that can change signaling pathways controlling proliferation, differentiation and apoptosis. Such mutations would be passed on to all of the stem cell's progeny, allowing evolution towards malignancy.

Recent studies have revealed that CSCs produce high levels of anti-apoptotic proteins and growth factors that make them refractory to antineoplastic treatments. The inefficacy of conventional therapies towards the stem cell population might explain cancer chemoresistance and the high frequency of relapse shown by the majority of tumors.

The possibility to isolate and study CSCs represents a revolutionary approach in cancer research. Indeed, these cells are responsible for the development of the tumor and represent the only cell population able to sustain tumor growth and progression. Therefore, CSCs represent the elective target for new targeted therapies, endowed with high and selective toxicity towards the tumor but harmless towards normal cells.

Evidence for the existence of CSCs was obtained first in the context of acute myeloid leukaemia [25] and thereafter in breast [26], colon [27], brain [28], prostate, ovarian tumors [29] and melanoma [30]. Important progress in human colon carcinoma has been achieved in the bio-pathology lab of this cooperative group characterizing CSCs as CD133 cells [31,32] and showing that these cultured cells retain the cancer-initiating potential upon injection into immuno-deficient mice. In addition, single cell cloning of these cells revealed that a single cell contains all the information necessary to reconstitute this tumor.

If tumor growth and metastasis are driven by a small population of CSCs, which like normal stem cells are relatively slow cycling, it may explain the failure of conventional therapies directed at killing rapidly dividing cells, which make up the remainder of the tumor. This new view of tumorigenesis has been challenging the approach to therapy since the emergence of recurrence is thought to result from the escape of CSCs from conventional anti-cancer therapies, designed to target the fast cycling and highly proliferating cancer cells. Tumors have been widely described to evade death signals generated by therapeutic drugs through the development of effective anti-apoptotic mechanisms, but the molecular bases of chemotherapy failure have not yet been defined in the majority of tumors. One particularly intriguing property of CSCs is that they are highly resistant to drugs and toxins because of the expression of several ABC transporters and anti-apoptotic factors and an active DNA-repair capacity. Therefore, the selective targeting of these cells appears necessary to eradicate tumors and prevent their recurrence. Moreover, a better understanding of CSCs role in the metastatic cascade may lead to novel therapeutic strategies against metastatic cancer.

CSCs can receive stimulatory signals from paracrine or autocrine factors that trigger death resistance abolishing thus the benefit of therapeutic interventions. The selectively hitting of these cells with specific drugs would in future allow the eradication of tumor preventing metastasis formation. The laboratories involved in the project are among the first one worldwide that isolated CSCs and developed the essential technologies for handling and studying them. These resources will be applied within the project to expand CSCs from selected tumors both *in vitro* and *in vivo* and carry out phenotypic and molecular characterization studies.

Another important feature of CSCs is the capacity to form a tumour, reproducing the parental tumour phenotype, when injected in immunodeficient mice and this characteristic makes them the best candidate for preclinical studies. For this reason during this project we will use an experimental model of xenotransplantation of tumor stem cells, which will allow to test new compounds on human-derived tumors grown in experimental animals.

3.0-STUDYOBJECTIVES

3.1 - Primary end-point

- To evaluate the feasibility of the project in clinical practice.

3.2 - Secondary end-points

- To identify LC, CRC and BC stem cells.
- To investigate the sensitivity to anti-tumor agents *in vitro*.
- To identify drugs potentially effective for a specific patient

4.0–PATIENTSSELECTIONCRITERIA

4.1 - Inclusion Criteria

- Histologically/cytologically confirmed diagnosis of metastatic NSCLC, CRC and BC.
- Availability of tumor tissue suitable for CSCs extraction
- Performance status of 100% according to Karnofsky score.
- Failure of conventional therapies or no therapy of proven efficacy.
- Adequate hematological, renal and liver functions.
- No concomitant comorbidity potentially interfering with the study.
- Informed consent form signature.

- If female: childbearing potential either terminated by surgery, radiation, or menopause, or attenuated by use of approved contraceptive method (intrauterine contraceptive device (IUD), birth control pills, or barrier device) during and for three months after trial

4.2 - Exclusion Criteria

- No possibility to obtain fresh tumor tissue.
- Performance status <100% according to Karnofsky score.
- Patient suitable for standard therapies.
- Important comorbidity interfering with the study.
- Significant alteration of liver, hematological or renal function(s).
- No informed consent form signature.

5.0- STUDY DESIGN

This is a prospective study assessing feasibility of individualized therapy in LC, CRC and BC patients. LC, CRC and BC patients with good performance status and tumor tissue collected before study enrollment, at failure of conventional therapies or without possibility to be treated with therapy of proven efficacy, will be considered eligible for the study. Tumor tissue will be collected before study entry, i.e tissue obtained during a diagnostic or therapeutical procedures, like surgery or biopsies with other purposes than the protocol. In vitro tumor sensitivity to chemotherapy drugs will be tested on tumor cell cultures per each patient. Drugs and their combinations will be considered effective if they kill ≥ 60% of tumor stem cells in vitro test.

6.0-EXPERIMENTALPROCEDURES

6.1 - Patients

Patients with LC, CRC and BC with good performance status and with available tumor tissue, at failure of conventional therapies or without possibility to be treated with therapy of proven efficacy, will be included in the study. Initial patient characterization will include detailed information on the following items: gender, age, clinical history, co-morbidity, physical examination, blood cell counts, complete blood analyses for liver and renal function, coagulation,

serological tumor markers, tumor location and stage, tumor mass, metastases.

6.2 - LC, CRC and BC CSCs identification

Isolation and characterization of CSCs will be made starting from samples of tumor tissue obtained from patients with NSCLC, CRC and BC obtained before study inclusion. The surgical/bioptical samples collected will be classified according to the specific histological characteristics of the tumor. From each sample, by means of enzymatic and mechanical procedures, the CSCs will be obtained and then cultivated in adequate culture mediums to be subsequently used for biochemical and molecular studies. Each sample will be associated with the patient history at the surgical time and an appropriate follow-up program consisting of periodic clinical and instrumental controls that in order to assign a prognostic value to the biological characteristics of the CSCs. Stem cells derived from the selected epithelial tumors will then undergo analysis of surface and intracellular markers in order to provide a definitive characterization of cellular phenotype. Stem cells derived from lung cancer, CRC and BC are identified as a subset of tumor cells positive for the marker CD133.

Tumor specimens will be washed several times and left over night in DMEM:F12 medium supplemented with high doses of Penicillin/Streptomycin and Fungizone in order to avoid contamination. Tissue dissociation will be carried out by enzymatic digestion and recovered cells cultured in serum-free medium containing 25 μg/ml insulin, 100 μg/ml apo-transferrin, 10 μg/ml putrescine, 0.03 mM sodium selenite, 20nM progesterone, 0.6% glucose, 5mM hepes, 0.1% sodium bicarbonate, 0.4% BSA, glutamine and antibiotics, dissolved in DMEM-F12 medium and supplemented with 20 ng/ml EGF and 10 ng/ml bFGF. Flasks non-treated for tissue culture will be used in order to reduce cell adherence and favourite growth of undifferentiated tumour-spheres. These culture conditions select for immature tumour cells, while non malignant or differentiated cells are negatively selected as assessed for CSCs of different origin [31]. Surviving immature tumor cells slowly proliferate giving rise to tumour cell aggregates, “spheres”, within 1-2 months in these culture conditions. Sphere-forming cells can be expanded by mechanical dissociation of spheres, followed by re-plating of single cells and residual small cell aggregates in complete fresh medium.

Differentiation of NSCLC, CRC and BC sphere-forming cells will be obtained by cell culture in specific medium (Cambrex). Phenotype of NSCLC, CRC and BC spheres and their differentiated progeny will be analyzed by flow cytometric analysis or immunofluorescence. In particular stem cell markers such as CD133, CD34 and BCRP1 will be analyzed.

Successively, cancer spheres will be analyzed in order to define the status of pathways involved in the process of proliferation, self-renewal and survival. In particular, tumor-specific analysis will be carried out to investigate the activity and the possible alteration of pathways responsible for stem cell homeostasis and global analysis (phosphoproteomic and signal transduction analysis, innovative drug testing, analysis of processes metastatization in vivo) aimed to provide an overall picture of the activation state of the key cellular pathways.

6.3 - Preclinical model

By using cancer spheres we will generate **orthotopic xenograft models** that recapitulate the parental tumor behaviour, including the aggressive features and the invasiveness potential. Orthotopic injection technique will be assessed in 5 weeks-old NOD/SCID mice. The injection procedure will be done with the support of a dissecting microscope. After anesthetization, 200 to 500 cancer sphere cells, modified in order to express a bioluminescent protein such as luciferase will be injected using a Hamilton syringe and 32-gauge needle. Metastatic and local tumors will be compared for their stem cell content through phenotypic analysis such as growth rate, or other stem cell properties including clonogenic capacity in soft agar or through limiting dilution assays. Infection of CSCs with lentiviral vector, coding for green fluorescent (GFP), as well as luciferase reporter proteins, will allow CSCs tracking *in vivo*. Particularly, the amphotropic packaging cell line 293T will be transfected by the calcium-phosphate/chloroquine method. Culture supernatants containing viral particles will be collected after 48h of transfection. Infection will be performed by culturing target cells in 0.45 μm filtered viral supernatant for 3h in a CO_2 incubator. Two infection cycles will be performed to infect cells. Microscopic evaluation of GFP expression in viral packaging and target cells will be performed by direct observation of cells using a reversed fluorescence microscope equipped

with a FITC filter. After infection, cells transduced with luciferase will be sorted by flow cytometry to obtain a pure marked population. The local tumor and the invasiveness development will be monitored through whole-body imaging techniques, that will permit to detect, localize and quantify *dynamically* the optical signal - bioluminescence - in a non invasive localization of the marked cell population. This procedure will be performed using the Photon imager *in vivo* imaging system (Biospace Lab), assisted by the most recent software for acquisition and image analysis. Thanks to this system, it was recently acquired in the laboratory and characterized by a very high sensitivity and 20 ms temporal resolution, we will analyze non anesthetized and freely moving animals. The bioluminescence signal will be acquired simultaneously as a standard video of the animal. Once we are sure of the success of tumour growing, mice will be sacrificed. Tumor will be removed for morphological characterization and phosphoproteomic analysis. This latter will be performed through reverse phase protein microarray, which allows the achievement of a high degree of sensitivity, precision and linearity, making possible to quantify the phosphorylated status of signal proteins in immature and differentiated lung cancer cells. This system will provide information on specific molecular pathways involved in NSCLC, CRC and BC cell growth and spreading.

6.4 - Tumor sensitivity to anti-tumoral agents

To selectively discriminate the effective therapeutic compounds against the putative tumor and invasion initiating cells, we will measure the viability of clonogenic LC, CRC and BC after the exposure to several anti-tumor drugs differentially combined at singular time point up to 96 hours. Priority on choosing drugs groups to be tested first will be defined according to histological and biological characteristics of the primary tumor.

7.0-PARTICIPATINGCENTERS

For the present study, patients will be enrolled at the Medical Oncology Department of Ospedale Civile in Livorno-Italy. Laboratory procedures will be performed at Fondazione Maugeri in Pavia and at the Laboratory of Cellular and Molecular Pathophysiology in Palermo.

8.0-STUDYDURATION

Patients required for the study will be enrolled in a 3 months period. A total of 20 patients will be enrolled. A total of 20 patients will be enrolled.

Bibliography

1. American Cancer Society. Cancer facts & figures 2010. Atlanta: American cancer society. 2010
2. Schiller JH, Harrington D, Belani CP, Langer C, Sandler A, Krook J, Zhu J, Johnson DH; Eastern Cooperative Oncology Group. Comparison of four chemotherapy regimens for advanced non-small-cell lung cancer. *N Engl J Med*. 2002 Jan 10;346(2):92-8
3. Zhou C, Wu Y-L, Chen G et al. OPTIMAL (CTONG 0802) study comparing first-line erlotinib versus carboplatin (CBDCA) plus gemcitabine (GEM), in Chinese advanced non small-cell lung cancer (NSCLC) patients (pts) with EGFR activating mutations. *Ann. Oncol*. 21(Suppl. 8), viii6 (2010) (Abstract LBA13)
4. Mok TS, Wu YL, Thongprasert S, Yang CH, Chu DT, Saijo N, Sunpaweravong P, Han B, Margono B, Ichinose Y, Nishiwaki Y, Ohe Y, Yang JJ, Chewaskulyong B, Jiang H, Duffield EL, Watkins CL, Armour AA, Fukuoka M. Gefitinib or carboplatin-paclitaxel in pulmonary adenocarcinoma. *N Engl J Med*. 2009 Sep 3;361(10):947-57.
5. Lee JS, Park K, Kim SW, et al. A randomized phase III study of gefitinib (IRESSATM) versus standard chemotherapy (gemcitabine plus cisplatin) as a first-line treatment for never-smokers with advanced or metastatic adenocarcinoma of the lung. *J Thor Oncol*. 2009;4(Suppl):PRS.4
6. Maemondo M, Inoue A, Kobayashi K, Sugawara S, Oizumi S, Isobe H, Gemma A, Harada M, Yoshizawa H, Kinoshita I, Fujita Y, Okinaga S, Hirano H, Yoshimori K, Harada T, Ogura T, Ando M, Miyazawa H, Tanaka T, Saijo Y, Hagiwara K, Morita S, Nukiwa T; North-East Japan Study Group. Gefitinib or chemotherapy for non-small-cell lung cancer with mutated EGFR. *N Engl J Med*. 2010 Jun 24;362(25):2380-8
7. Mitsudomi T, Morita S, Yatabe Y, Negoro S, Okamoto I, Tsurutani J, Seto T, Satouchi M, Tada H, Hirashima T, Asami K, Katakami N, Takada M, Yoshioka H, Shibata K, Kudoh S, Shimizu E, Saito H, Toyooka S, Nakagawa K, Fukuoka M; West Japan Oncology Group. Gefitinib versus cisplatin plus docetaxel in patients with non-small-cell lung cancer harbouring mutations of the epidermal growth factor receptor (WJTOG3405): an open label, randomised phase 3 trial. *Lancet Oncol*. 2010 Feb;11(2):121-8

8. Kwak EL, Bang YJ, Camidge DR, Shaw AT, Solomon B, Maki RG, Ou SH, Dezube BJ, Jänne PA, Costa DB, Varella-Garcia M, Kim WH, Lynch TJ, Fidias P, Stubbs H, Engelman JA, Sequist LV, Tan W, Gandhi L, Mino-Kenudson M, Wei GC, Shreeve SM, Ratain MJ, Settleman J, Christensen JG, Haber DA, Wilner K, Salgia R, Shapiro GI, Clark JW, Iafrate AJ. Anaplastic lymphoma kinase inhibition in non-small-cell lung cancer. *N Engl J Med*. 2010 Oct 28;363(18):1693-70
9. Scagliotti GV, Parikh P, von Pawel J et al. Phase III study comparing cisplatin plus gemcitabine with cisplatin plus pemetrexed in chemotherapy-naïve patients with advanced-stage non-small-cell lung cancer. *J Clin Oncol.* 26(21): 3543-51.2008.
10. Vamvakas L, Agelaki S, Kentepozidis NK et al. Pemetrexed (MTA) compared with erlotinib (ERL) in pretreated patients with advanced non-small cell lung cancer (NSCLC): Results of a randomized phase III Hellenic Oncology Research Group trial. *ASCO Meeting Abstracts* Jun 14, 2010: 7519.
11. Hanna N, Shepherd FA, Fossella FV et al. Randomized Phase III Trial of Pemetrexed Versus Docetaxel in Patients With Non–Small-Cell Lung Cancer Previously Treated With Chemotherapy. *J Clin Oncol* 22(9): 1589-97. 2004
12. Hurwitz H, Fehrenbacher L, Novotny W, Cartwright T, Hainsworth J, Heim W, Berlin J, Baron A, Griffing S, Holmgren E. Bevacizumab plus irinotecan, fluorouracil, and leucovorin for metastatic colorectal cancer. *N Engl J Med.* 2004; 350(23):2335–2342
13. Kabbinavar F, Hurwitz HI, Fehrenbacher L, Meropol NJ, Novotny WF, Lieberman G, Griffing S, Bergsland E. Phase II, randomized trial comparing bevacizumab plus fluorouracil (FU)/leucovorin (LV) with FU/LV alone in patients with metastatic colorectal cancer. *J Clin Oncol.* 2003; 21(1):60–65
14. Kabbinavar FF, Schulz J, McCleod M, Patel T, Hamm JT, Hecht JR, Mass R, Perrou B, Nelson B, Novotny WF. Addition of bevacizumab to bolus fluorouracil and leucovorin in first-line metastatic colorectal cancer: results of a randomized phase II trial. *J Clin Oncol.* 2005; 23(16):3697–3705
15. Saltz LB, Clarke S, Diaz-Rubio E, Scheithauer W, Figer A, Wong R, Koski S, Lichinitser M, Yang TS, Rivera F. Bevacizumab in combination with oxaliplatin-based chemotherapy as first-line

therapy in metastatic colorectal cancer: a randomized phase III study. *J Clin Oncol.* 2008; 26(12):2013–2019

16. Giantonio BJ, Catalano PJ, Meropol NJ, O'Dwyer PJ, Mitchell EP, Alberts SR, Schwartz MA, Benson AB 3rd. Bevacizumab in combination with oxaliplatin, fluorouracil, and leucovorin (FOLFOX4) for previously treated metastatic colorectal cancer: results from the Eastern Cooperative Oncology Group Study E3200. *J Clin Oncol.* 2007; 25(12):1539–1544.
17. Van Cutsem E, Köhne CH, Láng I et al. Cetuximab Plus Irinotecan, Fluorouracil, and Leucovorin As First-Line Treatment for Metastatic Colorectal Cancer: Updated Analysis of Overall Survival According to Tumor KRAS and BRAF Mutation Status. *J Clin Oncol*. May 20, 2011:2011-2019
18. Meads C, Round J, Tubeuf S et al. Cetuximab for the first-line treatment of metastatic colorectal cancer. *Health Technol Assess*. 2010 May;14 Suppl 1:1-8.
19. Plosker GL, Keam SJ et al. Trastuzumab: a review of its use in the management of HER2-positive metastatic and early-stage breast cancer. *Drugs*. 2006;66(4):449-75.
20. Cameron D, Casey M, Oliva C et al. Lapatinib plus capecitabine in women with HER-2-positive advanced breast cancer: final survival analysis of a phase III randomized trial. *Oncologist*. 2010;15(9):924-34.
21. Goldhirsch A, Colleoni M, Gelber RD. Endocrine therapy of breast cancer. *Ann Oncol*. 2002;13 Suppl 4:61-8
22. Dent R.A., et al., Safety and efficacy of the oral PARP inhibitor olaparib (AZD2281) in combination with paclitaxel for the first- or second-line treatment of patients with metastatic triple-negative breast cancer: Results from the safety cohort of a phase I/II multicenter trial. *J Clin Oncol (Meeting Abstracts)*, 2010. 28(15_suppl): p. 1018
23. FDA begins process to remove breast cancer indication from Avastin label. *http://www.fda.gov/newsevents/newsroom/pressannouncements/ucm237172.htm*
24. Pardal R, Clarke MF and Morrison SJ, Applying the principles of stem-cell biology to cancer. Nat Rev Cancer, 2003; 3(12):895-902.
25. Bonnet D, Dick JE. Human acute myeloid leukemia is organized as a hierarchy that originates from a primitive hematopoietic cell. *Nat Med*. 1997; 3(7):730-7

26. Dontu G, Jackson KW, McNicholas E, Kawamura MJ, Abdallah WM, Wicha MS. Role of Notch signaling in cell-fate determination of human mammary stem/progenitor cells. *Breast Cancer Res*. 2004;6(6):R605-15.
27. Lombardo Y, Scopelliti A, Cammareri P, Todaro M, Iovino F, Ricci-Vitiani L, Gulotta G, Dieli F, de Maria R, Stassi G. Bone morphogenetic protein 4 induces differentiation of colorectal cancer stem cells and increases their response to chemotherapy in mice. *Gastroenterology*. 2011;140(1):297-309.
28. Ricci-Vitiani L, Pallini R, Biffoni M, Todaro M, Invernici G, Cenci T, Maira G, Parati EA, Stassi G, Larocca LM, De Maria R. Tumour vascularization via endothelial differentiation of glioblastoma stem-like cells. *Nature*. 2010; 468(7325):824-8.
29. Bapat SA, Mali AM, Koppikar CB, Kurrey NK. Stem and progenitor-like cells contribute to the aggressive behavior of human epithelial ovarian cancer. Cancer Res. 2005 Apr 15;65(8):3025-9.
30. Fang D, Nguyen TK, Leishear K, Finko R, Kulp AN, Hotz S, Van Belle PA, Xu X, Elder DE, Herlyn M. A tumorigenic subpopulation with stem cell properties in melanomas. *Cancer Res*. 2005 Oct 15;65(20):9328-37.
31. Ricci-Vitiani L, Lombardi DG, Pilozzi E, Biffoni M, Todaro M, Peschle C, De Maria R. Identification and expansion of human colon-cancer-initiating cells. *Nature*. 2007 Jan 4;445(7123):111-5.
32. Todaro M, Alea MP, Di Stefano AB, et al. Colon cancer stem cells dictate tumor growth and resist cell death by production of interleukin-4. *Cell Stem Cell*. 2007 ;1(4):389-402.

Table 1. Drugs used for experimental assays

Chemotherapuetic Agents	Target agents
Cisplatin	Erlotinib
Carboplatin	Cetuximab
Vinorelbine	Sorafenib
Gemcitabine	Sunitinib
Paclitaxel	Bevacizumab
Docetaxel	Everolimus
Pemetrexed	Lapatinib
Adriamycin	Trastuzumab
Epirubicin	
5Fluorouracil	
Irinotecan	
Topotecan	
Dacarbazine	
Capecitabine	
Ciclofosfamide	
Vinblastina	
Mupharan	
Oxaliplatino	
Temozolomide	
Etoposide	

APPENDIX I - Karnofsky performance status scale

GRADE (%)	STATUS
100	Normal, no complaints, no evidence of disease
90	Able to carry a normal activity: minor signs or symptoms of disease
80	Normal activity with effort: some signs or symptoms of disease
70	Cares for self: unable to do normal activity or to do active work
60	Requires occasional assistance but is able to care for most of his/her needs
50	Requires considerable assistance and frequent medical care
40	Disabled: requires special medical care and assistance
30	Severely disabled: hospitalization is indicated although death is not imminent
20	Very sick: hospitalization necessary, active supportive treatment necessary
10	Moribund: fatal processed
0	Death

Osservatorio Nazionale sulla Sperimentazione Clinica dei Medicinali

La presente pagina di copertina, da allegare obbligatoriamente alla richiesta iniziale di autorizzazione all'Autorità competente e di parere del Comitato etico, contiene il Numero unico EudraCT assegnato alla sperimentazione clinica e costituisce la ricevuta dell'inserimento telematico dei dati nell'OsSC.

Numero EudraCT
2011-003421-10

**Richiedente**:<br>ASSOCIAZIONE ONCOLOGICA TRASLAZIONALE (AOT)		
**Codice protocollo:**<br>LIVONCO 2011/001	**Versione del protocollo:**<br>1.0	**Data del protocollo:**<br>11/08/2011
**Titolo completo della sperimentazione:**<br>STELLA: Studio di fattibilita' su un test di sensibilita' delle cellule staminali		

**Centro coordinatore / Centro clinico ***	
OSPEDALE LIVORNO - LIVORNO (LI)<br>SOTTOCOMITATO ETICO PER LA SPERIMENTAZIONE CLINICA DEI FARMACI DELLA AUSL 6 DI LIVORNO	**Disciplina:**<br>*ONCOLOGIA MEDICA*
**Sperimentatore coordinatore / Sperimentatore principale *:**<br>DR. FEDERICO CAPPUZZO<br><br>* In caso di sperimentazione monocentrica	

Osservatorio Nazionale sulla Sperimentazione Clinica dei Medicinali

This cover page has to be imperatively attached to the initial application for authorization to the competent Authority and for the opinion of the Ethical Committee. It contains the unique Number EudraCT given to the clinical research program and represents the confirmation of the electyronic data entry in the OsSC.

EudraCT Number

2011-003421-10

**Applicant:**<br>ASSOCIAZIONE ONCOLGICA TRASLAZIONALE (AOT)		
**Protocol Number:**<br>LIVONCO 2011/001	**PROTOCOL VERSION:**<br>1.0	**Protocol date:**<br>11/08/2011
**Full title of the research program:**<br>STELLA: Feasibility study on a chemosensitivity test on stem cells		

**Coordinator centre/ Clinical centre***	
LIVORNO HOSPITAL – LIVORNO (LI)<br>ETHICS REVIEW BOARD FOR INVESTIGATION OF DRUGS OF THE AUSL6 OF LIVORNO	**Classification:**<br>MEDICAL ONCOLOGY
**Coordinator researcher/Principal investigator*:**<br>DR FEDERICO CAPPUZZO<br><br>*In case of single site study	

Translation from Italian of the registration in the OsSC system

AZIENDA USL-6 DI LIVORNO

DIPARTIMENTO DI ONCOLOGIA MEDICA
Direttore: Dr. Federico Cappuzzo
U.O. ONCOLOGIA MEDICA

Direttore:
Dr. Federico Cappuzzo

Vice-Direttore:
Dr. Carmelo Tibaldi

Dirigenti Medici:
Dr. Andrea Antonuzzo
Dr.ssa Cecilia Barbara
Dr. Maurizio Bertuccelli
Dr.ssa Simona Bursi
Dr.ssa Samanta Cupini
Dr.ssa Ermelinda De Maio
Dr.ssa Roberta Di Marsico

Medici Specializzandi:
Dr. Manolo D'Arcangelo
Dr. Gabriele Minuti
Dr.ssa Armida D'Incecco

Medici Borsisti:
Dr.ssa Lorenza Landi

Responsabile C.O.R.D:
Dr.ssa Roberta Di Marsico

Responsabile Day Hospital:
Dr.ssa Samanta Cupini

Responsabile Degenza:
Dr.ssa Simona Bursi

Responsabile Trials Clinici:
Dr.ssa Ermelinda De Maio

<u>Gruppo patologia toracica</u>
Dr.Carmelo Tibaldi
Dr.ssa Cecilia Barbara

<u>Gruppo patologia mammaria</u>
Dr.ssa Ermelinda De Maio
Dr.ssa Roberta Di Marsico
Dr.ssa Simona Bursi

<u>Gruppo patologia gastrointestinale</u>
Dr.ssa Cecilia Barbara
Dr.ssa Simona Bursi
Dr.ssa Samanta Cupini

<u>Gruppo altre patologie</u>
Dr. Andrea Antonuzzo
Dr.Maurizio Bertuccelli

Data Management:
Dr.ssa Sara Fabiani

Coordinatrice Infermieristica :
AFD Antonella Dal Canto

Degenza:
0586 223416- 3284

Day Hospital:
0586 223458- 3344

Segreteria Libera Professione:
0586 253253

Fax:
0586 223457

LETTERA INFORMATIVA PER IL MEDICO CURANTE

Egregio Dottore/Gentile Dottoressa,

Con la presente desideriamo informarLa che il/la Suo/a assistito/a, Sig./Sig.ra ……………………………………………………………………………è stato inserito/a, con il consenso scritto dello/a stesso/a, nello studio clinico di fattibilità STELLA (**St**em C**ell**s Sensitivity **A**ssay), in quanto affetto da una neoplasia (polmonare, mammaria o colica) in fase avanzata, già trattata con tutti i regimi chemioterapici convenzionali e attualmente senza alternative terapeutiche di comprovata efficacia.

Il protocollo di studio prevede la valutazione *in vitro* in un laboratorio dedicato alla ricerca sulle cellule staminali tumorali, della loro sensibilità a farmaci chemioterapici e combinazioni. Recenti studi *in vitro* ed in cavie animali hanno dimostrato come le cellule staminali tumorali siano onnipotenti e siano il comparto cellulare della neoplasia e delle metastasi che sostiene la crescita e la progressione tumorale. L'attuale tecnologia ci permette di isolare e riprodurre *in vitro* le cellule staminali tumorali da campioni di tessuto tumorale, permettendo di testare la loro sensibilità a diversi agenti antitumorali in un breve periodo di tempo. In tal modo si prospetta la possibilità di identificare e offrire un trattamento individualizzato a pazienti con tumore polmonare, colo-rettale o mammario. Inoltre utilizzando le sferule di cellule staminali tumorali saranno generati modelli ortotopici che ricapitolano il comportamento del tumore di origine, inclusi le caratteristiche di aggressività ed il potenziale invasivo.

Lo studio STELLA è uno studio di fattibilità, ossia tendente a valutare le tempistiche e le eventuali problematiche relative alla procedura descritta in precedenza e quale possa essere, pertanto, la sua applicabilità nella pratica clinica.

La informiamo che il protocollo di studio STELLA, ad oggi da ritenersi sperimentale, è stato approvato dal Comitato Etico competente e verrà svolto secondo le linee guida internazionali e la normativa vigente in materia di ricerca clinica.

La ringraziamo sin d'ora per la collaborazione e restiamo a disposizione per eventuali chiarimenti.

Cordiali saluti

____ Firma

__________________________ Data

____ Recapito telefonico

INFORMATION LETTER FOR THE GENERAL PRACTITIONER

Dear Doctor,

With this letter we would like to inform you that your patient, Mr/Mrs/Miss…………………………………………………., has been included in the feasibility study STELLA (**St**em C**ell** Sensntivity **A**ssay) after signature of the informed consent form, because he/she has an advanced stage cancer (lung, breast or colorectal cancer), has already received all conventional chemotherapeutic treatments and no standard treatment options of proven efficacy are currently available for him/her. The study protocol includes the *in vitro* evaluation of sensitivity to anticancer single agents or combinations in a laboratory dedicated to cancer stem cells research. Recently, both *in vitro* and *in vivo* studies showed that cancer stem cells are omnipotent and represent the cell compartment that actually sustains tumor growth and progression. Current technology makes possible to isolate and propagate *in vitro* the cancer stem cells, starting from samples of tumor tissue and allows to testing their *in vitro* sensitivity against several anticancer agents in a short period of time. In this way, we anticipate the possibility of identifying and offering an individualized treatment to patients with lung, breast or colorectal cancer. Moreover, using the cancer stem cells spherules we will generate orthotopic models, which recapitulate the initial tumor as to behavior, aggressiveness and invasive potential.

The STELLA study is a feasibility study, i.e. evaluates timing and eventual issues of the above-described procedure, and whether this procedure can be applied to a clinical setting.

We inform you that the STELLA trial protocol has to be regarded to as experimental, it was approved from the local Ethical Committee and it will be developed according to international guidelines and the current legislation about clinical research.

Thank you for your collaboration and do not hesitate to contact us for further clarification.

Best regards

____ __________________________

Signature Date

____

Telephone number

Azienda USL-6 di Livorno

Dipartimento di Oncologia Medica
U.O. Oncologia Medica
Direttore: Dr. Federico Cappuzzo

Direttore:
Dr. Federico Cappuzzo

Vice-Direttore:
Dr. Carmelo Tibaldi

Dirigenti Medici:
Dr. Andrea Antonuzzo
Dr.ssa Cecilia Barbara
Dr. Maurizio Bertuccelli
Dr.ssa Simona Bursi
Dr.ssa Samanta Cupini
Dr.ssa Ermelinda De Maio
Dr.ssa Roberta Di Marsico

Medici Specializzandi:
Dr. Manolo D'Arcangelo
Dr. Gabriele Minuti
Dr.ssa Armida D'Incecco

Medici Borsisti:
Dr.ssa Lorenza Landi

Responsabile C.O.R.D:
Dr.ssa Roberta Di Marsico

Responsabile Day Hospital:
Dr.ssa Samanta Cupini

Responsabile Degenza:
Dr.ssa Simona Bursi

Responsabile Trials Clinici:
Dr.ssa Ermelinda De Maio

Gruppo patologia toracica
Dr.Carmelo Tibaldi
Dr.ssa Cecilia Barbara

Gruppo patologia mammaria
Dr.ssa Ermelinda De Maio
Dr.ssa Roberta Di Marsico
Dr.ssa Simona Bursi

Gruppo patologia gastrointestinale
Dr.ssa Cecilia Barbara
Dr.ssa Simona Bursi
Dr.ssa Samanta Cupini

Gruppo altre patologie
Dr. Andrea Antonuzzo
Dr.Maurizio Bertuccelli

Data Management:
Dr.ssa Sara Fabiani

Coordinatrice Infermieristica :
AFD Antonella Dal Canto

Degenza:
0586 223416- 3284

Day Hospital:
0586 223458- 3344

Segreteria Libera Professione:
0586 253253

Fax:
0586 223457

Lettera informativa

Gentile paziente,

Il Suo medico pensa che la partecipazione al protocollo di studio STELLA (**St**em C**ell**s Sensitivity **A**ssay) sia adatta per Lei, in quanto Lei è affetto/a da una patologia che è progredita dopo l'utilizzo dei farmaci comunementeimpiegati. Il Suo medico Le propone di procedere ad un saggio di sensibilità in vitro a diversi farmaci antitumorali sulle cellule staminali tumorali isolate da tessuto tumorale già precedentemente raccolto. I farmaci a cui queste cellule risulteranno più sensibili, saranno utilizzati per trattare la Sua malattia e garantirLe una ulteriore opzione terapeutica.

Cosa sono le cellule staminali tumorali?

Le cellule staminali tumorali sono una parte delle cellule che compongono la neoplasia e che contribuiscono al suo sostentamento. Le cellule staminali sono cellule normalmente presenti negli organismi viventi, che garantiscono il rinnovamento delle cellule dell'organismo stesso. Quando alcune mutazioni del DNA avvengono nelle cellule staminali, queste cominciano a riprodursi senza controllo e danno origine al tumore. Una parte delle cellule del tumore continuano ad avere le caratteristiche staminali e sostengono la crescita e la progressione del tumore. Sembra che colpire direttamente le cellule staminali tumorali con farmaci chemioterapici a cui esse risultino sensibili possa essere un modo per ottenere un buon risultato terapeutico.

Come saranno testati i farmaci chemioterapici?

Le cellule staminali tumorali isolate e riprodotte saranno utilizzate per testare la loro sensibilità in vitro ad una serie di farmaci chemioterapici e loro combinazioni.

Qual è lo scopo dello studio?

Scopo dello studio è valutare se la procedura sopradescritta può essere fattibile nella pratica clinica. Saranno valutate la percentuale di successo dell'isolamento delle cellule staminali ed i tempi necessari per il test di chemio sensibilità in vitro. Nel caso in cui lo studio dimostri che la procedura è fattibile, valuteremo mediante un altro studio l'impatto che tale strategia possa avere nel trattamento dei pazienti nella pratica clinica.

Cosa succede se si decide di non partecipare più allo studio?

La partecipazione allo studio STELLA è volontaria e Lei potrà comunicare al Suo medico la volontà di non partecipare più allo studio in qualsiasi momento, senza ripercussioni.

Tutela della privacy

Le informazioni relative al Suo stato di salute saranno gestite in maniera strettamente riservata sia dal medico responsabile della ricerca sia dal suo gruppo di collaboratori.

Informazioni circa i risultati dello studio

Se Lei lo richiederà, alla fine dello studio potranno esserLe comunicati i risultati dello studio in generale ed in particolare quelli che Le riguardano.

Non esiti a rivolgere al medico che La segue qualunque domanda riguardo lo studio e la terapia farmacologica proposta. Consideri anche l'idea di discutere l'argomento con un familiare o un amico.

Qualunque la partecipazione allo studio dovesse comportare dei danni a Suo carico, correlati alla partecipazione, Lei sarà risarcito in accordo con la normativa vigente: lo Sponsor ha stipulato a tal fine un'idonea copertura assicurativa con la compagnia ___________________________ (n° polizza____________________) di cui è conservata una copia presso il centro dove viene seguito e di cui potrà prendere visione qualora lo ritenga opportuno. La copertura assicurativa copre inoltre il costo di eventuali trattamenti medici, anche successivi alla sua partecipazione, qualora si verificassero delle complicanze causate dallo studio stesso.

Ulteriori informazioni

Per qualunque domanda, richiesta di chiarimento o problema, non esiti a contattare il Suo medico ai recapiti seguenti:

Dott. ____ Tel. ____

Dott. ____ Tel. ____

Indirizzo ____

____

MODULO DI CONSENSO INFORMATO

Il/La sottoscritto/a Nome ……………………………………………… Cognome ………………………………………………………………..

Nato a…………………………………………………………..Prov.…………………………in data…………………………………………………

Residente a……………………………………Prov…………………Via/piazza……………………………………CAP………………………

Affetto da ………

Dichiara

- di aver ricevuto copia del foglio informativo e modulo di consenso informato
- di aver letto e compreso i contenuti del foglio informativo e del modulo di consenso informato
- di aver avuto tempo sufficiente per riflettere sulle informazioni che sono state fornite e per porre domande al riguardo
- che sono state rese note le informazioni circa le caratteristiche, le finalità e le modalità di trattamento dei miei dati personali ai sensi del D.L. 196/2003 "Codice in materia di protezione dei dati personali"

Presta quindi consapevolmente e volontariamente il proprio consenso a partecipare al protocollo di studio STELLA (Stem Cells sensitivity Assay).

____

Numero di cartella clinica del paziente

____

Firma del paziente e data

Dichiara di aver discusso questo foglio informativo e consenso informato con il mio paziente, fornendo tutte le spiegazioni e chiarimenti richiesti.

____

Nome e cognome del medico che ha raccolto il consenso

____

Firma del medico che ha raccolto il consenso e data

INFORMATION LETTER

Dear Patient,

Your oncologist thinks that participation in the STELLA (**St**em C**ell** Sensitivity **A**ssay) study is ideal for you, because you have a tumor that has progressed on standard treatments. Your oncologist is proposing to perform an *in vitro* assay on the cancer stem cells isolated from your tumor and test them against several anticancer drugs. The most active drugs on these cells will be eventually used to treat your disease, thus providing further therapeutic options.

What are the cancer stem cells?

Cancer stem cells are part of the cells forming the tumor and contributing to its sustenance. Stem cells are normally present in our body and guarantee the renewal of tissues. Some DNA mutations may occur in these cells and, consequently, they start reproducing without control, leading to tumor formation. Part of the tumor cells preserve these stem-like features and sustain tumor growth and progression. It seems that selective targeting of cancer stem cells with chemotherapeutic agents to which they are sensitive can lead to a good therapeutic outcome.

How will we test the drugs?

Isolated and propagated cancer stem cells from your tumor will be tested *in vitro* for their sensitivity to anticancer single agents and combinations.

What is the objective of the study?

The principal aim of this study is to evaluate whether the procedure is feasible in clinic. We will evaluate the percentage of cases with successful isolation and the time frame for the *in vitro* chemosensivity assay. If the study shows that the procedure is feasible, we will evaluate the impact of this strategy on the treatment outcome in a successive clinical trial.

What happens if you no longer wish to participate in this study?

The participation in the STELLA study is voluntary and you are free to tell your oncologist that you no longer wish to participate in this study at any point with no consequences for you.

Privacy policy

Your health information will be managed from both the Principal Investigator of the research program and his collaborators in strict confidence.

Information on the results of the study

If you wish, results could be communicated to you at the end of the study, in particular those regarding yourself.

Do not hesitate to ask you oncologist questions about the study and the pharmacologic therapy he/she will propose. Take in consideration the idea of discussing your participation with relatives and/or friends.

If participation in the study causes you any damage directly connected with the study, you will obtain an indemnity according to the current legislation. The sponsor insured the study with the company___________________ (Policy #___________________). A copy of the policy is stored in the centre where you are followed. You can ask to view it whenever you wish. The insurance policy covers also the cost of eventual medical treatment caused by your participation in the study.

Further information

Do not hesitate to contact your oncologist for any question, clarification or issue at the follwing numbers:

Dr____ Tel____________________

Dr____ Tel____________________

Address____

INFORMED CONSENT FORM

Forename……………………………………………………Surname………………………….………………

Place of birth……………………………………….Date of birth………………………………………………

Resident in……………………………Address………………………………ZIP code………………………..

Affected with……

I declare that:

- I received a copy of the information letter and informed consent form;
- I read and understood the contents of the information letter and informed consent form;
- I had sufficient time to think about the info I received and to ask questions;
- I was informed on the characteristics, aims and management of my sensitive data (D.L. 196/2003 “Codice in materia di protezione dei dati personali”).

Therefore, I consciously and voluntarily consent to participate in the STELLA (Stem Cells Sensitivity Assay) study.

Number of clinical record

Patient’s signature and date

I declare that I discussed this information letter and informed consent form with my patient, giving him/her all the info and explanation he/she needed.

Forename and surname of the oncologist who collected the informed consent

Signature of the oncologist who collected the informed consent and date
